# Genetic polymorphisms of collagen type I α1 chain (*COL1A1*) gene increase the frequency of low bone mineral density in the subgroup of children with juvenile idiopathic arthritis

**DOI:** 10.1186/1878-5085-4-15

**Published:** 2013-06-13

**Authors:** Mikhail M Kostik, Arseniy M Smirnov, Grigory S Demin, Marina M Mnuskina, Larisa A Scheplyagina, Valentina I Larionova

**Affiliations:** 1 Hospital Pediatric Department, Saint-Petersburg State Pediatric Medical University, Lytovskaya 2, Saint-Petersburg 194100, Russia; 2 Gene, Ltd, Saint-Petersburg 194156, Russian Federation; 3 Genetic systems, Ltd, Saint-Petersburg 195027, Russian Federation; 4 Department of biochemistry, Diagnostic Center#1 for adults, Saint-Petersburg, 194354, Russian Federation; 5 Moscow scientific and research clinical institute named M.F, Vladimirskiy, Moscow 129110, Russian Federation; 6 Department of molecular diagnostics, Turner’s Scientific and Research Institute for Children's Orthopedics, Saint-Petersburg 196603, Russian Federation

**Keywords:** Juvenile idiopathic arthritis (JIA), Bone mineral density (BMD), Bone densitometry, Collagen, Polymorphism

## Abstract

**Background:**

Collagen type I is one of the key proteins involved in the maturation, development and mineralization of bone. Genetic polymorphisms of collagen type I alpha-1 chain (*COL1A1*) gene are associated with low bone mineral density and higher risk of fractures in adults and children. We hypothesize that the polymorphic alleles and genotypes of *COL1A1* gene influence bone mineralization and metabolism in children with juvenile idiopathic arthritis (JIA).

**Methods:**

We recruited 196 children with JIA in our study. Bone mineral density (BMD) was measured by lumbar spine dual-energy X-ray absorptiometry. Osteocalcin, Ca, Ca^2+^ and inorganic phosphate (Pi) were utilized for the assessment of bone metabolism. Molecular testing: Sp1 (rs1800012) and -1997G/T (rs1107946) polymorphisms of *COL1A1* gene were detected RFLP.

**Results:**

No differences in genotype, allele and haplotype distribution of *COL1A1* were detected among children with normal and low BMD (LBMD; <−2 standard deviation). The presence of GG genotype of Sp1 increased the incidence of LBMD in Tanner II to III children (odds ratio (OR) = 9.7 [95% confidence interval (CI), 1.2; 81.7], *p* = 0.02) as well as GG genotype of -1997G/T increased the frequency of LBMD in Tanner IV to V children (OR = 4.5 [95% CI, 0.9; 22.0], *p* = 0.048). Tanner I children with -1997GG genotype had lower Ca^2+^ and osteocalcin and higher Pi compared with carriers of -1997Т allele. Tanner IV to V children with -1997GG genotype had lower BMD and BMD-Z score than carriers of -1997Т.

**Conclusions:**

The evaluation of the biologic effects of the GG Sp1 and GG of -1997G/T polymorphism of *COL1A1* has shown negative effect on BMD and mineral turnover related to pubertal stage.

## Overview

Children with juvenile idiopathic arthritis (JIA) have disturbances in bone mineralization which are realized in the lack of bone mineral accrual, growth delay and fractures [1-4]. In JIA patients, factors that impact bone metabolism include the subtype of arthritis, disease duration, concomitant treatment with systemic steroids and disease activity [5,6]. However, there is no strict relationship between negative factors that impact bone mineralization and bone damage [5,7]. Some patients quickly decrease bone mineralization, realize fractures and do it very quickly, while others are still resistant to negative factors that influence bone health [8,9]. Indeed, the rate of bone loss or lack of bone mineral gain depends on the combination of environmental (disease and physical activity, diet) and genetic factors involved in bone metabolism. The knowledge about the factors associated with increased bone mineral loss may help to select appropriate treatment and preventive program in JIA patients with high risk of bone damage [10-15]. There are only few studies about the possible role of genetic factors in bone metabolism [16]. Unfortunately, reported studies are difficult to compare due to the differences in the studied population, subgroups of arthritis, disease duration, concomitant treatment and definition of bone health damage. Difficulties in the interpretation of the prognostic role of genetic factor in the prevention of bone damage in JIA patients lead to these genetic factors to be rarely studied. One of the well-studied and clinically significant genetic factors that influence bone mineralization is collagen type I.

Collagen type I is one of the key proteins associated with bone quality, strength and health, because this is a main protein of the bone organic matrix involved in bone maturation, development and mineralization [17]. The most striking example is osteogenesis imperfecta, in which mutations in genes, encoding different types of collagen invoke more than 90% of patients, lead to low bone mineralization and frequent fractures [18]. Not only mutations but also genetic polymorphisms of collagen type I alpha-1 chain (*COL1A1*) gene are also associated with low bone mineral density and higher risk of fractures in adults and children [19,20]. The same genotypes influence on the risk of ligaments and tendon injuries, joint dislocations and formation of lumbar disk disease [21-23]. Sp1 polymorphism is located in the intronic part of *COL1A1* gene, associated with transcription start site, and -1997G/T polymorphism is located in promoter region of this gene. These polymorphisms affect on DNA banding affinity of transcription factors involved in bone metabolism, such as Nmp4, Osterix and Sp1. Functional activity of polymorphic genotypes is associated with increased transcription activity and enhanced collagen synthesis that leads to misbalance in normal alpha-1 and alpha-2 chain ratio (2:1) of collagen type I and realized in disturbances of bone mineralization and fall of bone strength [24]. Normally, carriers of SS (GG) genotype have normal alpha-1 and alpha-2 chain ratio (1.99±0.07 to 1.0) of collagen type I, but heterozygous have increased alpha-1 and alpha-2 chain ratio (2.36±0.1 to 1.0). Excessive synthesis of alpha-1 chains leads to the formation of molecules of collagen consist of three alpha-1 chains - heterotrimers [α1(I)_3_], characterized by abnormal precipitation of inorganic salts. Carriers of Ss (GT) genotype have less inorganic and more organic components of bone that affect bone strength and mineralization [25]. Bone biopsy exposed that GT carriers had lower mineralization and impressive heterogeneity compare with GG carriers of Sp1 *COL1A1*. *In vitro* studies have revealed low osteoblast activity to form sites of bone mineralization in GT carriers which also affects bone strength [26].

The objective of the present study was to investigate the role of Sp1 and -1997G/T genetic polymorphisms of the *COL1A1* gene in bone mineralization and turnover in children with JIA.

The biggest meta-analysis which covered 24,511 participants including results of 32 studies shows that TT genotype of *COL1A1* Sp1 polymorphism is associated with the modest reduction of hip (0.16 units, *p* = 1 × 10^−6^) and lumbar spine (LS) bone mineral density (BMD) (0.13 units, *p* = 0.01) and increased risk of fractures (1.31-fold, *p* = 0.02) in the whole group and in the subgroup of females as compared to GG carriers. No differences in males were observed. Borderline significance was observed between LS BMD of -1997GG homozygotes and carriers of the T allele (GT+TT). In females, LS BMD values were lower (0.06 units, *p* = 0.02) in the GG homozygotes as compared with G/T heterozygotes. No associations with genotypes and BMD in males and no association with fracture risk in whole group and subgroups were observed [27]. The results in the studies included in this meta-analysis were controversial.

The presence of ss (TT) genotype was associated with low BMD without increased fracture rate in Greek postmenopausal women [28] and in French premenopausal women [29]. Braga et al. in a group of perimenopausal and postmenopausal women showed that ss genotype is associated with the lowest BMD levels in hip and LS, but they also determined the age-related effect of polymorphic genotypes. The effect of *COLIA1* was not visible until the sixth decade of life, but it increased thereafter with aging, becoming statistically significant also at the lumbar spine in subjects aged >70 years. The authors suggest that this polymorphism is more related with age-dependent bone loss than with peak bone mass [30].

Both reduced BMD and increased risk of fractures were observed in ss genotype carriers in several studies. Keen et al. have shown association of ss genotype with the reduction of LS BMD, increased risk of fractures and increased urinary pyridinoline [31]. McGuigan et al. did not find any significant difference in bone density, rate of bone loss, body weight, height or years since menopause between the *COL1A1* genotype groups but have revealed that s allele is an independent predictor of fractures only in women. The incidence of fractures was 45 times higher in those with low BMD who carried ‘s’ allele [32]. In the study of Bernad et al., the frequency of TT (ss) genotype was significantly higher in osteoporotic postmenopausal women than in non-osteoporotic and in osteoporotic women with fractures compare with non-fracture females, odds ratio (OR) for fractures = 5.9 for TT compared with GG+GT genotypes [33]. The similar results were observed in a group of 1,044 75-year-old Swedish women. The presence of at least one copy of the ‘s’ allele was associated with lower femoral neck (FN) BMD and increased risk of wrist fractures: OR=2.73 for the ss homozygotes and 1.4 for the Ss heterozygotes when compared with the SS homozygotes [34].

The results of some studies did not find any association of polymorphic genotypes with BMD in the group of postmenopausal women [35,36] in pre- and perimenopausal women [37].

In contrast, some studies have not revsealed relations between Sp1 *COLIA1* and lumbar spine and femoral neck BMD in Irish females before and after menopause [38], in UK females elder than 75 years in bone mineralization and fractures prevention [39] and in Chinese males and females [40]. In 100 Chinese, Sp1 polymorphism was not found, and the authors suggest that the absence of such relation is typical for Asians, described earlier [40].

In longitudinal 18-year study about the relation of BMD loss and Sp1 *COLIA1* polymorphic genotypes in postmenopausal females, there were no difference in lumbar spine and femoral neck BMD loss due to polymorphic genotypes. Patients with Ss and ss genotypes of Sp1 *COLIA1*, considering a lot of researches, have to have lower BMD, in practice, in this study had higher forearm BMD than SS genotype carriers, that is, controversial to the results of study described earlier. The authors didnot support the hypothesis that Ss *COLIA1* genotype predisposes to increased ranges of BMD loss and low BMD [41].

Opposite results were detected in patients with somatic diseases. In Hungarian patients with primary biliary cirrhosis and high percentage of osteoporosis, there was a lower frequency of s allele Sp1 *COLIA1* than in controls. There were no differences in mineralization in patients with polymorphic genotypes [42]. Perhaps, it is possible that due to the influence of pathogenic disease factors on BMD, and interactions between genes and disease.

Few pediatric studies show controversial results, as well as in adults. In a Norwegian study that included 269 healthy boys and girls over the 4 years old, there were no differences in mineralization with polymorphic genotypes, excluding a small group of boys with ss genotype Sp1 *COLIA1* who had higher femoral neck BMD compare with SS and Ss [43]. Possibly, it could be due to the different ages of children included in study, and separate analysis in children of equal age and pubertal development has not been performed. In the study carried out in Los Angeles, vertebra mineralization and size were evaluated in 109 prepubertal Mexican girls. Revealed changes are equal to the majority of adult's studies: girls with SS genotype Sp1 *COLIA1* had higher BMD than in girls that are carriers of s allele (Ss and ss). There were no significant differences in vertebra size in girls with polymorphic genotypes [44]. In the largest pediatric study, performed in UK, the analysis of the role of Sp1 *COL1A1* and Pvull *COL1A2* polymorphic genotypes on fracture risk (FR) was carried out separately, depending on gender, fracture site and pubertal stage. The presence of s allele Sp1 *COL1A1* was associated with increased FR (OR = 3.1 [95% confidence interval (CI) 1.43 to 6.61], *p* = 0.004) only in prepubertal children (Tanner I). In mid-pubertal and pubertal children, polymorphic genotypes did not relate with FR. Also, there were no differences in bone mineralization in children with polymorphic genotypes in any of studied groups [19]. In a group of 258 prepubertal Caucasian children from Australia, there were no associations between Sp1 binding site of the *COLIA1* polymorphic genotypes, and v-BMD of FN and LS were found [45].

Among Turkish children with *forma magna* of β-thalassemia and osteoporosis, the s allele of Sp1 *COLIA1* was more frequent than in controls (healthy adults without osteoporosis), but the highest BMD-Z score in the lumbar spine was in carriers of s allele (ss>Ss>SS). Opposite it, patients with SS genotype had the highest mineralization parameters in femoral neck, but the data were insignificant [46].

## Methods

### Patients and controls

Approval was obtained from the Saint-Petersburg State Pediatric Medical University committee on the ethics of research on human beings. Blood samples were obtained after informed written consent. All specimens for the labs were collected at the same time as the DNA specimens.

One hundred ninety-six children with JIA were enrolled in the study between January 2003 and December 2007. Each child was a patient of the Saint-Petersburg State Pediatric Medical University rheumatology clinic. All patients were Caucasians. We enrolled all patients who fulfilled the European League Against Rheumatism JIA criteria and agreed to take part in our study [47]. Only one child per family was enrolled. All patients were divided into three groups according to arthritis course: oligoarticular, polyarticular and systemic arthritis. Extended oligoarticular patients and systemic arthritis patients without signs of systemic activity were included in polyarticular group.

Exclusion criteria were children with any serious gastrointestinal problems, malabsorption, liver diseases, alimentary and other known types of dystrophy. Children who had received hormone replacement therapy (estrogens, oral contraceptive) or had disease known to affect bone metabolism (diabetes mellitus and others endocrinopathy, skeletal dysplasia, rickets-like diseases) and children with non-traumatic fractures in whom idiopathic osteoporosis could be suspected also were excluded. Supplementation of calcium and vitamin D was allowed only for children, who were treated by corticosteroids (CS). Non-CS children were excluded if they had calcium and vitamin D supplementation in the previous 3 months before study enrollment. No patients with biologic treatment were included in the present study.

### Assessment of bone mineralization and bone metabolism

Bone mineralization was measured in 196 patients by dual-energy X-ray absorptiometry of lumbar spine at L1-L4 (Hologic QDR 4500C densitometer with reference pediatric database, Hologic Inc., Bedford, MA, USA). Densitometry parameters, such as bone area (cm^2^), bone mineral content (g) and BMD (measured in g/cm^2^ and in Z score, standard deviation (SD)), were all evaluated. Low bone mineral density (LBMD) for chronological age was defined by Z score < 2 SD, according to the recommendation of the International Society for Clinical Densitometry 2007 [48].

For the assessment of bone metabolism the following measures were used: osteocalcin (bone gla-protein, a marker of osteosynthesis) using immunochemiluminiscent assay. Also, levels of total and ionized calcium (Ca^++^) and inorganic phosphate were determined.

### Genetic analysis

For the detection of *COL1A1* Sp1 (rs1800012) and -1997G/T (rs1107946) polymorphisms, the previously published method [49,50] was used. After the treatment of PCR product containing Sp1 polymorphism by Bse1I endonuclease following DNA fragments were generated: 63 and 22 bp for Sp1 G allele, 85 bp for Sp1 T allele. PCR product containing -1997G/T polymorphism was treated by BstMAI endonuclease whereupon following DNA fragments were generated: 275 and 58 bp for -1997G allele, 333 bp for -1997T allele. The size of DNA fragments was estimated using polyacrylamide gel electrophoresis. One hundred ninety-six and 188 patients were analyzed for Sp1 and -1997G/T polymorphisms, respectively.

### Statistical analysis

Associations between the *COL1A1* polymorphisms and bone mineralization and bone metabolic markers in JIA patients were analyzed using Statistica 6.0 (StatSoft, Tulsa, OK, USA). We utilized chi-square test, Fisher's exact test and Mann–Whitney analysis. Data are shown in median and quartiles (Me; 25%; 75%). Because we had a restricted number of JIA children with -1997ТТ and Sp1 TT genotypes for statistical analysis, all children were divided in two groups: carriers of the T allele (GT+TT genotypes) and carriers of GG genotype (no T allele). *P* values < 0.05 were considered to indicate a significant difference. No significant differences in anthropometry and disease activity status related to *COL1A1* polymorphisms were found.

## Results

The main demographic and clinical features of study patients were shown in Table 1. We had no differences in genotype distribution in the group with growth delay (linear growth <10th percentile) compare with JIA children with normal linear growth (25th to 75th percentile). Also, we compared genotype distribution between children with growth delay (<10th percentile) with children with increased linear growth (>90th percentile), and no differences were observed. Thus, these two polymorphic markers are not associated with linear growth in our cohort.

**Table 1:** Demographics of JIA patients included in the present study

**Parameters**	**JIA patients, *n *= 198**
Age, years^a^	12.0 (7.0; 15.0)
Girls/boys, *n* (%)	116 (58.6)/82 (41.4)
Height, cm^a^	154.5 (123.3; 165.0)
Height<10th%, *n* (%)	14 (7.1)
Weight, kg^a^	42.0 (25.0; 55.0)
Weight<10th%, *n* (%)	27 (13.6)
BMI^a^	
Moderate and severe thinness (BMI ≤ 17.0), *n* (%)	17.35 (15.6; 20.1)
Underweight (17.0 < BMI ≤ 18.5), *n* (%)	87 (43.9)
Normal (18.5 < BMI < 25.0), *n* (%)	35 (17.7)
Pre-obese (25.0 ≤ BMI < 30.0), *n* (%)	62 (31.3)
Obese (BMI ≥ 30.0), *n* (%)	12 (6.1)
Puberty stage, Tanner	2 (1.0)
Pre-pubertal (I)	71 (35.9)
Mid-puberty (II to III)	54 (27.3)
Pubertal (IV to V)	73 (36.9)
JIA course, *n* (%)	
Oligoarticular	112 (56.6)
Polyarticular	68 (34.3)
Systemic	18 (9.1)
Onset age, years^a^	7.0 (3.9; 12.0)
Disease duration, year^a^	2.0 (0.5; 2.0)
Active joints, *n*	2.0 (1.0; 6.0)
ESR, mm/h^a^	6.0 (4.0; 25.0)
CRP, mg\l^a^	5.0 (2.2; 18.0)
Glucocorticoides, *n* (%)	52 (26.3)
DMARD, *n* (%)	81 (40.9)
LBMD, *n* (%)	44 (22.2)
Fractures (vertebral and non-vertebral), *n* (%)	29 (14.6)

^a^Me (IQR).

The analysis of distribution of genotypes, alleles and haplotypes of *COL1A1* has not revealed differences among children with normal and LBMD. In pubertal subgroups, some differences were observed.

Prepubertal children (Tanner I) had changes of some parameters of bone metabolism and mineral turnover associated with -1997G/T *COL1A1* polymorphic alleles and genotypes. Children with -1997GG genotype compared with carriers of -1997Т allele had lower Ca^++^, osteocalcin and higher inorganic phosphate. The presence of -1997GG in prepubertal children was associated with lower bone turnover without the impact of bone mineralization and frequency of LBMD and fracture rate.

In early to mid-puberty children, i.e., Tanner stages II to III, the presence of the GG genotype of Sp1 Col1A1 leads to the increased incidence of LBMD. In pubertal children, who nearly completed growth, i.e., Tanner stage IV to V associations with other polymorphic genotypes were observed. Pubertal children with -1997GG genotype had lower BMD, BMD-Z score and increase frequency LBMD than carriers of -1997Т (Tables 2 and 3). No differences in parameters of bone metabolism and mineral turnover related to Sp1 polymorphic genotypes in pubertal subgroup were observed.

**Table 2:** JIA patients with Sp1 and -1997G/T polymorphic genotypes, divided according to LBMD and pubertal stage

**Population**	**Genotype**	**LBMD**	**NBMD**	**OR [95% CI], ‘GG’ baseline**	***p***
	*Sp1*				
All JIA patients (*n* = 196)	‘GG’	30 (69.8)	107 (69.9)		0.75
	‘GT’	13 (30.2)	44 (28.8)		
	‘TT’	0 (0.0)	2 (1.3)		
JIA patients in Tanner stages II to III (*n* = 54)	‘GG’	14 (93.3)	23 (59.0)	9.7 [1.2; 81.7], sensitivity −0.93,	0.02^a^
	‘GT’ and ‘TT’	1 (6.7)	16 (41.0)	Specificity - 0.41	
	*−1997G/T*				
All JIA patients (*n* = 188)	‘GG’	28 (66.7)	96 (65.7)		0.77
	‘GT’	12 (28.6)	46 (31.5)		
	‘TT’	2 (4.7)	4 (2.8)		
JIA patients in Tanner stages IV to V (*n* = 70)	‘GG’	12 (27.3)	2 (7.7)	4.5 [0.9; 22.0], sensitivity −0.27,	0.048
	‘GT’ and ‘TT’	32 (72.7)	24 (92.3)	Specificity - 0.92	

^a^Fisher's exact test.

**Table 3:** Parameters of bone metabolism, mineralization and mineral turnover related to pubertal stage and -1997G/T Col1A1 polymorphic genotypes

**Population**	**Parameter**	**‘GG’**	**‘GT’ and ‘TT’**	***p***
JIA patients in Tanner stage I (*n* = 68)	Ca^++^, mmol/l	1.12 (1.05; 1.17)	1.15 (1.1; 1.2)	0.03
	Pi, mmol/l	1.67 (1.56; 1.73)	1.6 (1.47; 1.63)	0.03
	osteocalcin, ng/ml	81.4 (66.8; 95.6)	108.3 (105.4; 120.4)	0.02
JIA patients in Tanner stage IV to V (*n* = 70)	BMD, g/cm^2^	0.84 (0.76; 0.93)	0.91 (0.88; 0.96)	0.03
	BMD-Z score, SD	−1.29 (−2.1; −0.57)	−0.65 (−1.14; 0.39)	0.009

We focused the association between *COL1A1* genotypes and bone mineral density and metabolism in the subgroup of children with JIA according Tanner stage. Disease activity, lack of locomotion and concomitant treatment are the main damage factors of bone health and linear growth in JIA children. In relation to bone status, the JIA population is quite heterogenous due to different arthritis subtypes, number of active joints, disease duration, dosage and kind of treatment, age of affected children and puberty status. Some JIA patients have increased bone sensitivity and susceptibility to negative factors related to age, puberty and perhaps, to genetic background. The sequence of bone mineral and metabolic disturbances due to puberty stage and genotype was observed in our study. Prepubertal children had only decreased rate of bone turnover without changes of BMD, but children with advanced Tanner stages had already decreased bone mineralization. The results of our study had some controversies compared to previous studies. Some different ethnic populations and differences in genotype distributions can provide the contrasts of results. In our study, GG (SS) genotype is associated with LBMD. Similar results were described before Berg et al. [47] and Guzeloglu-Kayisli et al. [50] in which S allele related with osteoporosis and low BMD. Comparison of genotype distribution has revealed significant differences between the results of our study and studies with similar results. The highest frequency of TT (ss) genotype was observed in the work of Berg et al. and the highest frequency of GG in Guzeloglu-Kayisliet al. The data of genotype distributions in previous and our studies are shown in Table 4. The results of previous studies do not give as a clear answer to the question which of the *COLIA1* polymorphism is more related to bone mass in both adult and children populations. Controversial results could be explained by the features of individuals (healthy or ill), racial and national differences, type of data analysis considering interfere of gender, age, pubertal stage and any others environmental factors which are accompanied with gene-gene interactions. The results of our study have limitations related to interaction of environmental and genetic factors and heterogeneity of studied population (age, puberty, disease parameters).

**Table 4:** **Comparison of the distribution of Sp1 genotypes of *COLIA1 *gene in different populations**

**Genotypes**	**Berg et al. 2000**	**Sainz et al. 1999**	**Blades et al. 2010**	**Tao et al. 1999**	**Guzeloglu-Kayisli et al. 2008**	**Our study**	***p***
GG (SS)	197 (73.2)	86 (78.8)	124 (67.0)	92 (66.7)	77 (84.0)	137 (69.9)	0.02
GT (Ss)	61 (22.7)	22 (20.1)	54 (30.0)	40 (29.0)	15 (16.0)	57 (29.1)	
TT (ss)	11 (4.1)	1 (0.9)	5 (3.0)	6 (4.3)	0 (0.0)	2 (1.0)	
*p* (vs. our study)	0.05	0.23	0.46	0.14	0.036	-	-

## Conclusions

The evaluation of biologic effects of GG Sp1 and GG of -1997G/T polymorphism of *COL1A1* has shown negative effect on bone mineralization and some markers of bone metabolism and mineral turnover related to pubertal stage.

1. The presence of GG genotype of Sp1 *COL1A1* increased the risk of LBMD in mid-puberty JIA children.

2. Pubertal children with -1997GG genotype *COL1A1*had lower BMD and BMD-Z score than carriers of -1997Т allele. The presence of -1997GG genotype increased the frequency of LBMD.

3. Prepubertal children with -1997GG genotype had lower ionized calcium, osteocalcin and higher inorganic phosphate as compared with carriers of -1997Т allele carriers.

## Expert recommendations

The testing of genetic polymorphism can detect the target population of children with JIA who are more susceptible to the development of bone mineral disorders. Also, we suggest that genetic polymorphisms can play the adaptive role in bone mineralization and turnover regulating into different age intervals.

## Abbreviations

BMD: Bone mineral density; CI: Confidence interval; COL1A1: Collagen type I alpha-1 chain; CS: Corticosteroids; DNA: Deoxyribonucleic acid; FR: Fracture risk; JIA: Juvenile idiopathic arthritis; LBMD: Low bone mineral density; LS: Lumbar spine; OR: Odds ratio.

## Competing interests

The authors declare that they have no competing interests.

## Authors’ contributions

MMK, AMS, GSD and MMM were responsible for the acquisition of data. AMS and GSD carried out the molecular genetic studies. MMM performed biochemistry tests. MMK did the literature search and the statistical analysis, and wrote the paper. LAS and VIL participated in study design and coordination and helped to draft the manuscript. MMK, LAS and VIL interpreted the data and were responsible for the manuscript preparation. All authors read and approved the final manuscript.
